# Polymeric immunoglobulin receptor suppresses colorectal cancer through the AKT-FOXO3/4 axis by downregulating LAMB3 expression

**DOI:** 10.3389/fonc.2022.924988

**Published:** 2022-08-05

**Authors:** Ding Zhang, Hao Huang, Ting Zheng, Lei Zhang, Binbin Cui, Yanlong Liu, Shiheng Tan, Liyuan Zhao, Tian Tian, Lijing Gao, Qingzhen Fu, Zesong Cheng, Yashuang Zhao

**Affiliations:** ^1^ Department of Epidemiology, School of Public Health, NHC Key Laboratory of Etiology and Epidemiology (23618504), Harbin Medical University, Harbin, China; ^2^ Department of Colorectal Surgery, The Third Affiliated Hospital of Harbin Medical University, Harbin, China

**Keywords:** colorectal cancer, polymeric immunoglobulin receptor, prognostic marker, AKT-FOXO3/4 axis, methylation-driven gene

## Abstract

Colorectal cancer (CRC) remains one of the most common malignancies worldwide and its mechanism is unclear. Polymeric immunoglobulin receptor (PIGR) which plays an important role in mucosal immunity is widely expressed in the mucosal epithelium and is dysregulated in different tumors. However, the role and underlying mechanisms of PIGR in CRC remain unclear. Here, we demonstrated that PIGR was hypermethylated and downregulated in our cohort (N = 272), and these features were associated with reduced overall survival in patients (HR_methylation_ 1.61, 95% CI [1.11-2.33]). These findings were validated by external TCGA and GEO data. Moreover, PIGR overexpression inhibits CRC cell malignant phenotypes *in vitro* and impedes CRC cells growth in male BALB/c nude mice. Mechanistically, PIGR physically associates with RE1 silencing transcription factor (REST) and blocks the transcription of laminin subunit beta 3 (LAMB3). Subsequently, the AKT-FOXO3/4 axis was suppressed by downregulated LAMB3. In the drug sensitive assay, PIGR-overexpressing cells were more sensitive to cisplatin and gemcitabine. Together, PIGR may serve as a powerful prognostic biomarker and putative tumor suppressor by suppressing the AKT-FOXO3/4 axis by downregulating LAMB3 in CRC. Our study may offer a novel therapeutic strategy for treating CRC patients who highly express PIGR with cisplatin and gemcitabine.

## Graphical Abstract

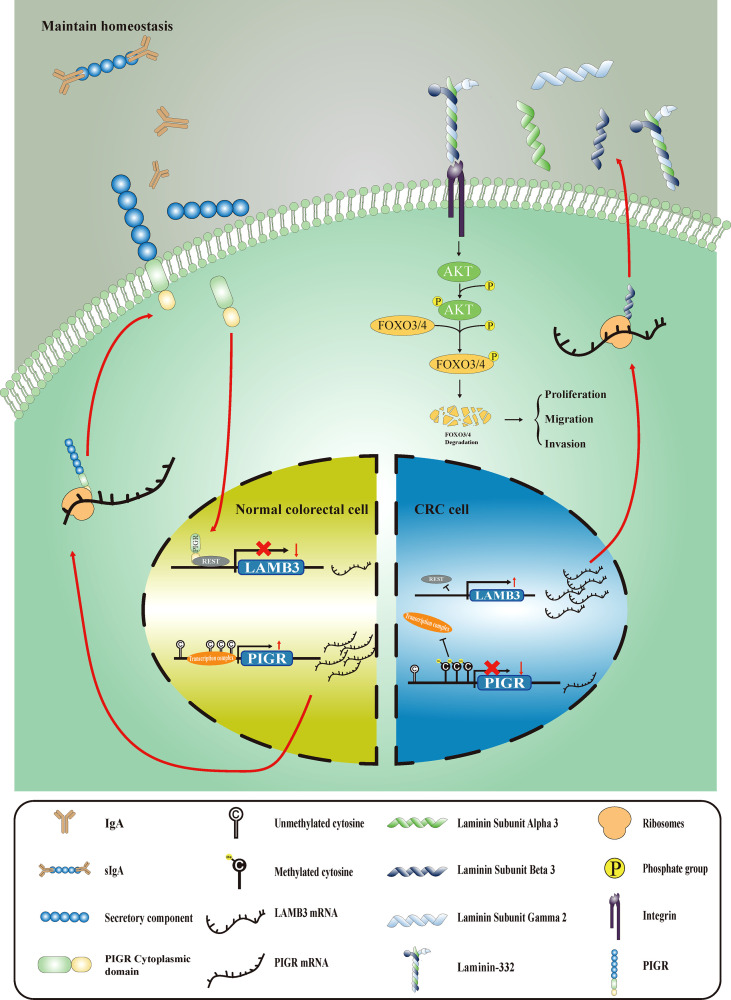

## Introduction

Colorectal cancer (CRC) is one of the most common malignant tumors worldwide, accounting for approximately 8.2% of new cases and 8.7% of new deaths in various types of cancers (1). Patients with early-stage CRC have a better prognosis; however, the 5-year survival rate rapidly drops to below 21% when patients have lymph node or distant metastasis (2). Therefore, it is important to reveal the molecular mechanisms of CRC progression, which provide information for the development of new preventative and therapeutic methods and improve the prognosis of CRC.

Epigenetic modifications, such as DNA methylation, are considered heritable alternations in gene expression but do not affect DNA sequences (3, 4). Aberrant DNA methylation is the most common epigenetic alteration in tumors. Hypermethylation of the promoter region is one of the mechanisms of tumor suppressor gene inactivation. In the 1980s, it was discovered that a tumor suppressor gene, retinoblastoma suppressor gene, was silenced by aberrant DNA methylation, providing strong evidence of the role of epigenetic alterations in tumorigenesis (5). Genes that exhibit expression that is significantly associated with their methylation status are called methylation-driven genes. Although studies using bioinformatic algorithms have identified the prognostic or diagnostic value of methylation-driven genes in breast cancer (6), lung cancer (7), liver cancer (8), and CRC (9), these studies did not explore the molecular mechanisms of these methylation-driven genes.

Polymeric immunoglobulin receptor (PIGR) is a member of the immunoglobulin superfamily. It specifically binds to dimeric IgA and pentameric IgM and transports them to the apical membrane through transcellular transport (10, 11). PIGR plays an important role in mucosal immunity, which is the first line of defense against infection. In colorectal tissues, loss of PIGR expression increases the risk of instability in the homeostasis of the intestinal environment (12). However, the role of PIGR in different tumors is inconsistent. PIGR is abnormally expressed in different tumors (13–17). Mechanistically, PIGR promotes tumor growth and metastasis in hepatocellular carcinoma (18, 19) and pancreatic cancer (20), whereas PIGR inhibits tumor proliferation in lung cancer (21). However, the role and mechanisms of PIGR in CRC remain unclear.

Hence, we systematically investigated the role and molecular mechanisms of the methylation-driven gene PIGR in CRC tumorigenesis.

## Material and methods

### Study subjects and biospecimen collection

A total of 279 colorectal cancer tissues and 27 adjacent noncancerous tissues were collected from The Third Hospital of Harbin Medical University from 2004 to 2012 after obtaining consent. We followed up postoperative patients at 3–6 months intervals for the first year after tumor resection and then once a year. The follow‐up ended in 2018. The survival time of patients was measured using overall survival (OS). The use of patient specimens followed the ethical principles of the Declaration of Helsinki.

### DNA extraction, bisulfite conversion and targeted bisulfite sequencing

Genomic DNA from tissue samples was extracted using the phenol-chloroform method. Then, purified DNA was bisulfited by a DNA modification kit (Qiagen, Hilden, Germany) according to manufacturer’s instructions.

The methylation levels of three CpG sites in *PIGR* promoter (CpG_1: 207120042, CpG_2: 207120023 and CpG_3: 207119988) were determined by MethylTargeted sequencing (Genesky Biotechnologies Inc.). The DNA fragments containing these three CpG sites were amplified, and sequenced on the Illumina platform. We filtered out samples with low target coverage (< 10×) in CpG sites and high missing rates (> 30%). Eventually, 306 samples remained for this study.

### Plasmid construction

The full-length coding sequence of PIGR, LAMB3, REST were cloned into expression vectors. The point mutation plasmids of PIGR were generated by DpnI method. LAMB3 promoter was cloned into a luciferase reporter vector. For more details, please see Supplementary Methods.

### Cell culture and cell transfection

RKO, HCT-8, LS-513 and LOVO cells were cultured in a humidified incubator at 37°C and 5% CO_2_ atmosphere in MEM, RPMI-1640, RPMI-1640 and F-12K, supplemented with 10% fetal bovine serum, 100 U/ml penicillin and 0.1 mg/ml streptomycin.

Cells were seeded in a six-well plate until the confluence reached about 80% to transfect. Transfection of plasmids were performed using Lipofectamine™ 2000 (Invitrogen) according to manufacturer’s instructions. Two days after transfection, the appropriate concentration of G418 was added into medium (700 μg/ml for RKO and 600 μg/ml for HCT-8) for about two weeks. Then, G418-resistant cells were screened and used for further assays.

### Extraction of total RNA and qRT-PCR

Total RNA was extracted using TRIzol (Invitrogen) reagent and cDNA was synthesized by ReverTra Ace^®^ kit (Toyobo, FSQ201) following the manufacturer’s instructions. QRT-PCR were performed by SYBR green mix (Toyobo, QKD-201) on a Roche LightCycler 480 instrument. Thermal Cycling conditions as follows: an initial denaturation step of 98°C for 2 minutes; 40 cycles at 98°C for 10 s and 60°C for 10 s and 68°C for 30 s; melting curve analysis. The amplification efficiency of all reactions was between 90% to 110%. Finally, the relative expression was calculated through 2^-ΔΔCt^ methods. The qPCR primers used for gene expression analysis are shown in **Table S7**.

### RNA-seq and bioinformatics analysis

Total RNA of RKO cells stably transfected with pCDNA3.1-PIGR and pCDNA3.1 (–) vector was sequenced by Bioacme Biotechnology Co., Ltd. (Wuhan, China). For more details, please see the Supplementary Methods. Differentially expressed genes (DEGs) between the overexpressed PIGR cell group and the control group were identified using the DESeq2 algorithm (22). An absolute fold-change value ≥ 2 and a false discovery rate of ≤ 0.05 were used as the criteria for identifying significant DEGs. The R package “clusterProfiler” was used to perform Kyoto Encyclopedia of Genes and Genomes (KEGG) enrichment analysis and gene set enrichment analysis (GSEA) (23).

### Prediction of the sensitivity of chemotherapy drugs

To evaluate the half-maximal inhibitory concentration (IC_50_) of chemotherapy drugs in the PIGR-overexpressing cells and control cell groups, we used the R package “pRRophetic” (24). By constructing the ridge regression model based on the Genomics of Drug Sensitivity in Cancer (GDSC) (http://www.cancerrxgene.org/) cell line expression spectrum and TCGA gene expression profiles, we used the pRRophetic algorithm to predict the IC_50_ of different drugs.

### Cell viability assay

Cell proliferation assay and drug sensitivity assay were performed by CCK-8 kit (Dojindo). RKO and HCT-8 cells (5 × 10^3^ or 4 × 10^3^ per well, respectively) were seeded into 96-well plates. For proliferation assay, cells were cultivated for 5 specific time points (0, 24, 48, 72 and 96 hours); for drug sensitivity assay, cells were cultivated for 24 hours and a series of concentration of cisplatin, gemcitabine, oxaliplatin and capecitabine were added into the medium and cultured for another 24 hours. Then, CCK-8 reagent was added and cultured for another an hour and then the absorbance was measured at 450 nm.

### Colony formation assay

For colony formation assay, 500 cells per well were seeded into six-well plates. After 10 days of culture, the cells were washed and fixed for 15 minutes. Then the cells were stained with crystal violet for 10 minutes. Greater than 50-cell colonies were counted for further statistical analysis.

### CRC cell line xenograft assay

10 male BALB/c nude mice (4 weeks of age) purchased from the Beijing Vital River Laboratory Animal Technology Co., Ltd., were housed in a standard pathogen-free environment. For subcutaneous mouse models, 3 × 10^6^ CRC cells (RKO-Vector or RKO-PIGR) which suspended in 100 μl of PBS buffer were subcutaneously injected into the right armpit of the mice. The mice were monitored every day. When the tumor became visible, the tumor was measured by a vernier caliper 3 times a week. Four weeks later, all mice were sacrificed. Tumor volume was calculated as follow: Length × Width^2^/2.

### Transwell assay

For migration assay, RKO and HCT-8 cells (5 × 10^4^) were starved for 12 hours and seeded into the top chamber of inserts with 8 μm pores (Corning, 3422), which supplied with serum-free medium, while proper medium contained 20% FBS was added into the lower chamber. Twenty-four hours later, cells were washed by PBS and fixed with absolute methanol for 15 minutes. Subsequently, cells were stained with crystal violet and non-migrating cells were gently removed by a cotton swab. Invasion assays were performed using inserts pre-coated with properly diluted Matrigel (BD Biosciences, 356234). Cells which moved to the bottom membrane were counted by imaging 4 randomly fields.

### Wound healing assay

RKO and HCT-8 cells were starved for 12 hours and seeded into six-well plates. The scratches were made by 200 μl sterile pipette tips when cells confluence reached 95%. Cells were cultured with proper serum-free medium and images were collected at three different time points (0, 24 and 48 hours).

### Western blot and immunoprecipitation

Total proteins of tissues and cells were extracted by RIPA buffer (Beyotime, P0013B) supplemented with 1% (v/v) 100 mM PMSF and phosphatase inhibitor (Roche, 4906837001). For immunoprecipitation, cells were lyses in non-ionic detergent RIPA buffer (Beyotime, P0013D) supplemented with 1% (v/v) 100 mM PMSF and phosphatase inhibitors (Roche, 4906837001). The Western blot and immunoprecipitation protocol is described in detail in the Supplementary Materials and Methods.

### Immunofluorescence

Cells were seeded on coverslips, washed three times with cold PBS buffer, and then fixed with absolute methanol for 10 min at -20°C. After washed in PBS for 10 min three times, cells were incubated in blocking buffer (PBS, 5% BSA) 1 hour at room temperature. The relevant primary antibodies diluted (1:500) in blocking buffer were added overnight at 4°C. Cells were washed with PBS buffer 10 min three times and incubated with CoraLite 488-conjugated (Proteintech, SA0013-2) or CoraLite 594-conjugated secondary antibodies (Proteintech, SA0013-4) at 1:500 dilution for 1 h at room temperature. After 3 × 10 min washes with PBS buffer, cells were stained with Hoechst (Beyotime, C1017), coverslips mounted with anti-fade mounting medium (Beyotime, P0126), and slides examined by fluorescence microscopy.

### Dual luciferase reporter assay

In brief, the recombinant plasmids pGL3 containing LAMB3 promoter and pGL4.75 were co-transfected into RKO and HCT-8 cells. Forty-eight hours later, luciferase activity was determined using the dual luciferase reporter assay kit (Promega, E1910).

### Statistical analysis

Continuous variables are reported as the means ± standard deviation. Based on the distribution of our data, Pearson and Spearman correlation tests, unpaired or paired Student’s t tests, Wilcoxon rank sum tests, one-way ANOVA, normality tests and Fisher’s exact tests were performed to analyze the differences between different groups. Kaplan–Meier curves were utilized to compare the survival rate between the different groups. Univariate and multivariate Cox regression models were applied to analyze prognostic factors. R version 4.1.1 software was used for statistical analyses. All the tests were two-tailed, and P < 0.05 was considered to be statistically significant.

### Data availability

The DNA methylation data, RNA-seq data and clinical information from two The Cancer Genome Atlas (TCGA) projects (COAD and READ) were downloaded from the UCSC (https://xena.ucsc.edu/). RNA expression data (GSE106582, GSE39582, GSE161158, GSE14333, GSE17536 and GSE17537) and methylation array data (GSE101764) of CRC samples were downloaded from the Gene Expression Omnibus (GEO, https://www.ncbi.nlm.nih.gov/geo/). The RNA-seq expression profiles of normal colon tissues were downloaded from the Genotype-Tissue Expression (GTEx) database. Quantitative protein expression data of CRC tissues from the Clinical Proteomic Tumor Analysis Consortium (CPTAC) database were downloaded from cBioPortal (http://cbioportal.org/). The targeted bisulfite sequencing and RNA-seq data generated during the current study are available from the corresponding author on reasonable request.

## Results

### The PIGR promoter is hypermethylated in CRC tissues and correlated with unfavorable prognosis

**Figure 1:**
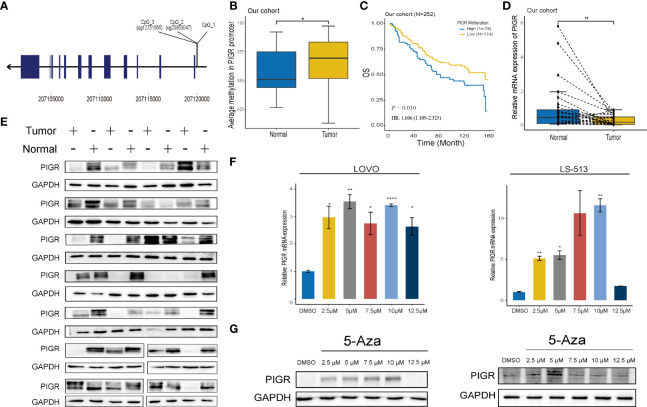
PIGR is downregulated and hypermethylated in CRC tissues and associated with CRC patients’ survival **(A)** The positions of 3 sequenced CpG in PIGR promoter. **(B)** The methylation of PIGR promoter in adjacent normal tissues (N = 27) and CRC tissues (N = 279) in our cohort. **(C)** Kaplan-Meier curve of OS based on the methylation of PIGR promoter in tumor tissues of our cohort. **(D)** The relative PIGR mRNA expression in matched 28 pairs of CRC tissues and adjacent normal tissues. **(E)** Western blot of the PIGR expression in CRC tissues and matched adjacent normal tissues. Kaplan-Meier curve of OS based on the methylation of PIGR promoter in tumor tissues of TCGA cohort. **(F)** The relative PIGR mRNA expression after treated with a series concentration of 5-Aza in LOVO and LS-513 cells (Differences compared to DMSO group) (n = 3; one-way ANOVA, *P* adjusted by FDR). **(G)** The PIGR protein expression after treated with a series concentration of 5-Aza in LOVO and LS-513 cells. *P < 0.05; **P < 0.01, ****P < 0.0001.

The overall workflow of this study is presented in **Figure S1**. To explore whether methylation of the PIGR promoter in CRC tissues was altered during CRC development, we first detected three CpG loci targeting the PIGR promoter region (**Figure 1A**) using targeting bisulfite sequencing in 279 CRC tumor tissues and 27 adjacent normal tissues in our cohort. We found that the methylation levels of three CpG loci were significantly increased in tumor tissues compared with adjacent normal tissues (**Figure 1B**) and that the methylation level was increased with tumor stage (**Figure S2A**). Kaplan–Meier curves and Cox regression showed that patients with a hypermethylated PIGR promoter tended to have a poor prognosis (**Figure 1C**; **Table S1**). However, multivariate Cox regression analysis indicated that PIGR methylation was not an independent prognostic factor of poor survival in our cohort (*P* = 0.064). In addition, aberrant methylation of PIGR was closely associated with distant metastasis (*P* = 0.005), tumor stage (*P* = 0.039) and OS status (*P* = 0.021) (**Table 1**).

**Table 1:** Association between PIGR methylation and clinicopathological features in CRC patients of our cohort and TCGA cohort.

Variables	PIGR methylation				PIGR methylation		
	(Our cohort)				(TCGA cohort)		
	High	Low	*P* Value		High	Low	*P* Value
**Age**							
<65	53	108	0.479		116	62	0.340
≥65	25	63			121	81	
**Sex**							
Female	28	70	0.486		102	70	0.288
Male	50	101			135	73	
**Tumor stage**							
Early	34	100	**0.039**		111	79	**0.050**
Advanced	44	71			117	54	
**Lymph invasion**							
No	37	98	0.166		118	87	**0.043**
Yes	40	70			117	55	
**Distant metastasis**							
No	69	166	**0.005**		162	99	0.083
Yes	8	3			39	13	
**Type**							
COAD	29	63	1.000		171	105	0.441
READ	49	108			57	28	
**OS Status**							
Alive	33	101	**0.021**		176	118	0.076
Dead	46	73			61	25	

The bold values mean the difference is statistically significant.

TCGA and GEO datasets were applied for external validation. Probes cg12751565, which corresponds to CpG_3, and cg20953047, which corresponds to CpG_2, of the 450k methylation array were chosen for validation. Likewise, in both TCGA and GEO datasets, the methylation level of the PIGR promoter was higher in CRC tissues (**Figures S2B**, **C**) and advanced stages (**Figure S2D**). The prognostic value of PIGR promoter methylation in the TCGA cohort was consistent with the results in our cohort (**Figure S3A**; **Table S2**).

### PIGR is downregulated in CRC tissues and correlated with poor prognosis

The qRT–PCR and western blot revealed that PIGR mRNA and protein expression was significantly decreased in CRC tissues compared with matched adjacent normal tissues (28 pairs) (**Figures 1D**, **E**). Then, TCGA and GEO datasets were used to validate thhe mRNA expression of PIGR, and CPTAC dataset was used to validate PIGR protein expression. These results showed that PIGR expression was reduced in CRC tissues (**Figures S2E**–**G**) and in advanced stages (**Figures S2H**, **I**).

In addition, patients who lost PIGR expression were associated with a poor prognosis based on several external datasets (**Figures S3B**–**G**; **Tables S3**–**5**).

### The methylation level of PIGR promoter is negatively correlated with its expression

We found that PIGR expression was negatively correlated with PIGR promoter methylation using TCGA and GEO data (r_TCGA_ = -0.759 and r_GEO_ = -0.740) (**Figures S2J**, **K**). To further validate the relationship between the methylation level of PIGR and its expression, we used 5-Aza (sigma) as a demethylation reagent. We chose LS-513 and LOVO cells for further experiments because they have a higher methylation level in PIGR promoter based on the Cancer Cell Line Encyclopedia database. After treating cells with a series of 5-Aza concentrations, the expression of PIGR mRNA and protein were elevating with the increasing concentrations of 5-Aza (**Figures 1F**, **G**).

### Overexpressed PIGR suppresses CRC cells proliferation, migration and invasion

**Figure 2:**
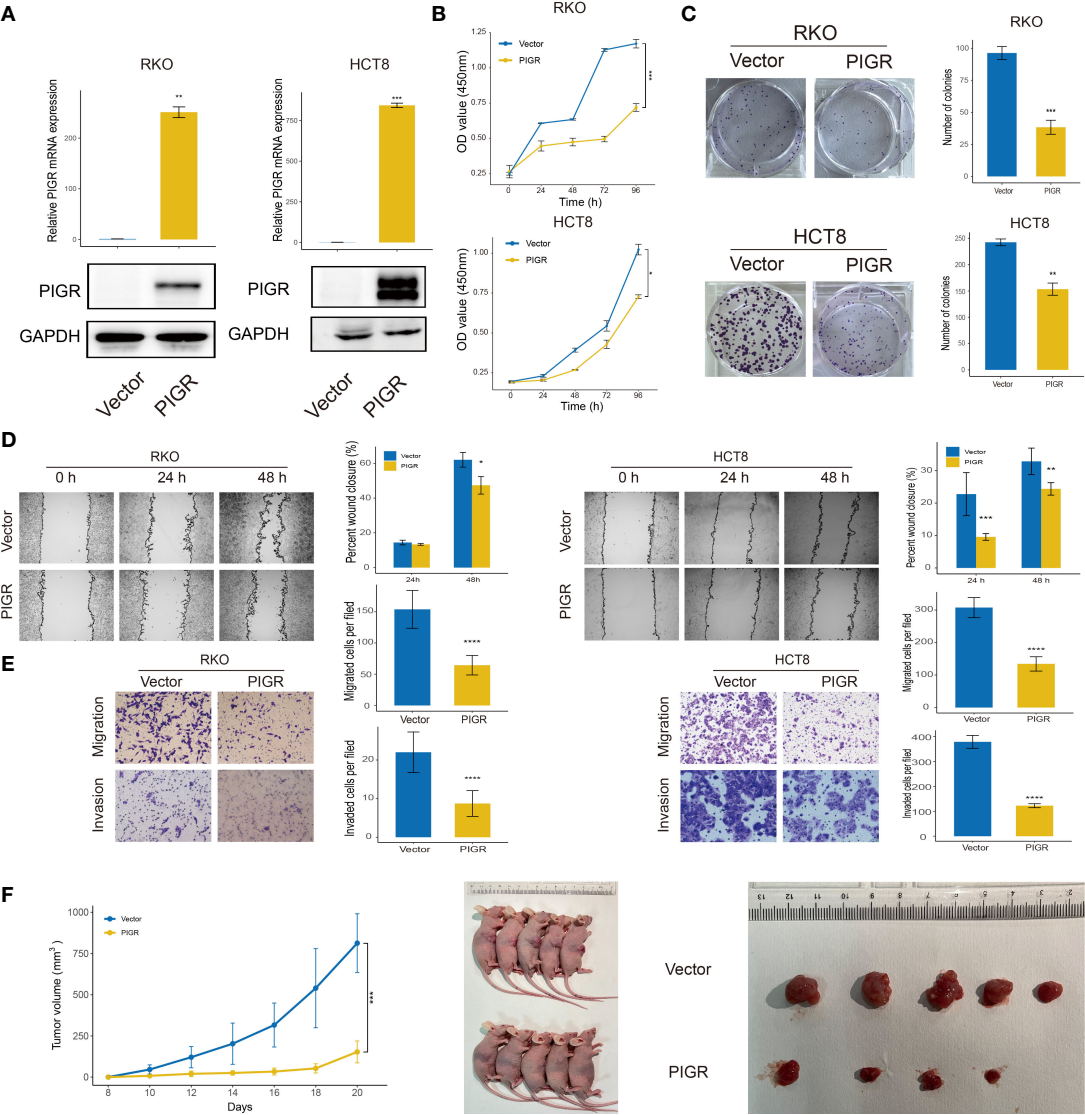
PIGR inhibits cell proliferation, migration and invasion **(A)** Overexpression of PIGR was confirmed by qRT-PCR and western blot in RKO and HCT-8 cells (n = 3; Student’s t-test). **(B)** Cell proliferation was detected by CCK-8 assay at 0, 24, 48, 72 and 96 hours in RKO and HCT-8 cells (n = 3; Student’s t-test). **(C)** The colony formation assay of RKO and HCT-8 cells (n = 3; Student’s t-test). **(D, E)** Wound healing, migration, and invasion assays after PIGR overexpression in RKO and HCT-8 cells (n = 3; Student’s t-test). **(F)** Effects of PIGR-overexpressing on RKO cells growth by establishment of subcutaneous xenograft mouse models. Tumor size and tumor growth curve are shown (n = 5; Student’s t-test). Data are presented as the mean ± S.D. *P < 0.05; **P < 0.01; ***P < 0.001; ****P < 0.0001.

RKO and HCT-8 cells were stably transfected with PIGR (**Figure 2A**). Our data showed that PIGR overexpression inhibited RKO and HCT-8 cells proliferation and migrations. In addition, Transwell assays with Matrigel also revealed that the invasion capability was significantly suppressed in PIGR-overexpressing cells (**Figures 2B**–**E**).

To further validate the role of PIGR in tumor growth *in vivo*, a xenograft assay was performed by subcutaneously injecting tumor cells into nude mice. Consistent with the results of the proliferation assay *in vitro*, we found that PIGR in RKO cells significantly inhibited tumor growth (**Figure 2F**).

### PIGR expression is inversely associated with LAMB3 expression

**Figure 3:**
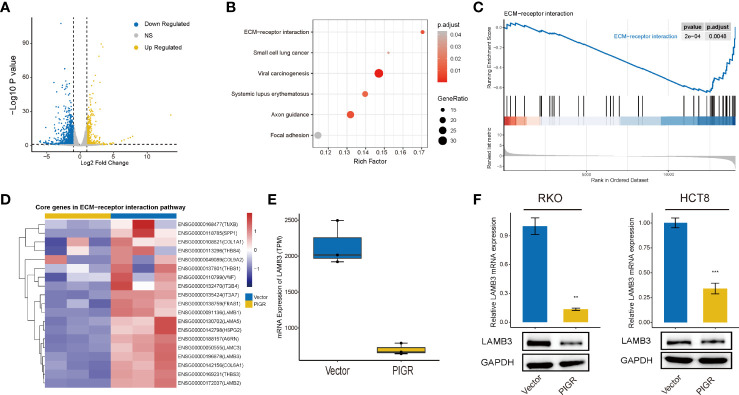
PIGR downregulates ECM-receptor interaction pathway and LAMB3 **(A)** Volcano plot showing the DEGs between stably transfected with empty vector and PIGR in RKO cells. **(B)** KEGG enrichment analysis of DEGs. **(C)** Enriched gene sets in ECM-receptor interaction pathway by the high PIGR expression. **(D)** Heatmap of core genes in ECM-receptor interaction pathway identified by GSEA. **(E)** Difference of LAMB3 mRNA expression between PIGR-overexpressing cells and control cells. **(F)** The mRNA and protein levels of LAMB3 in RKO and HCT-8 cells with PIGR-overexpressing were detected by qRT-PCR (n = 3; Student’s t-test). **P < 0.01; ***P < 0.001.

Then, we performed RNA-seq to identify the DEGs between PIGR-overexpressing RKO cells and control cells. We eventually obtained 557 upregulated genes and downregulated 1067 genes (**Figure 3A**).

KEGG enrichment analysis demonstrated that the genes were mostly enriched among the ECM (extracellular matrix)-receptor interaction, focal adhesion and small cell lung cell pathways (**Figure 3B**). GSEA revealed that the PIGR-overexpressing group downregulated more genes in ECM-receptor interaction pathways (NES = -1.935, *P*adj = 0.0048) (**Figure 3C**) than the control group. Therefore, we assumed that PIGR might play a tumor suppressor role in CRC by affecting cell adhesion and altering extracellular matrix components. Nineteen core genes in the ECM-receptor interaction pathway were identified (**Figure 3D**). We observed that laminin subunit beta 3 (LAMB3, one of the core genes) was significantly downregulated in PIGR-overexpressing cells compared with control cells (**Figure 3E**; **Figure S4A**). These results were further validated by qRT–PCR and western blot in two different CRC cell lines (**Figure 3F**).

### PIGR inhibits proliferation, migration and invasion by targeting LAMB3

**Figure 4:**
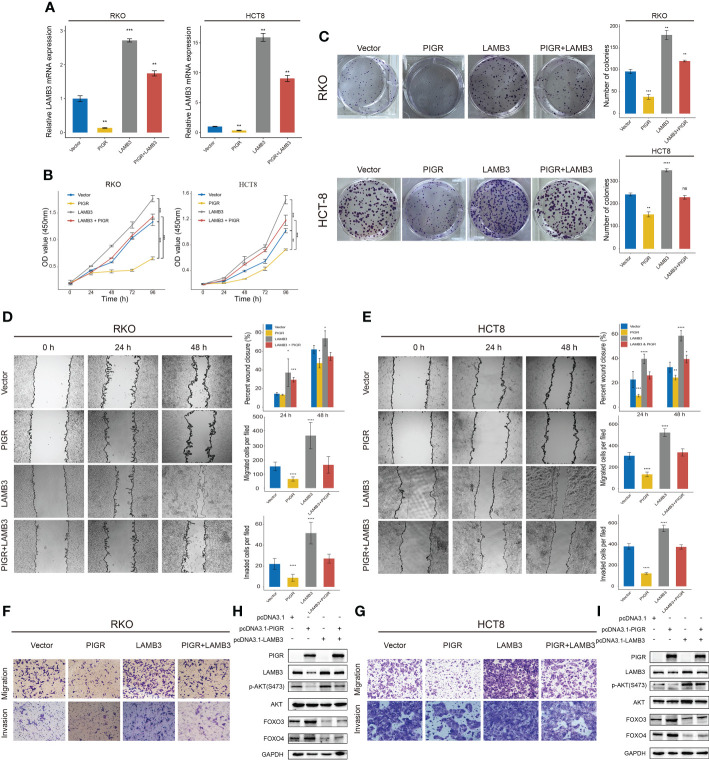
PIGR suppresses AKT-FOXO3/4 axis through LAMB3 **(A)** Relative LAMB3 mRNA expression of empty vector (Vector), PIGR-overexpressing (PIGR), LAMB3-overexpressing (LAMB3) and rescue experiment group (PIGR & LAMB3) in RKO and HCT-8 cells. Differences compared to Vector group (n = 3; one-way ANOVA, *P* adjusted by FDR). **(B)** Cell proliferation was detected by CCK-8 assay at 0, 24, 48, 72 and 96 hours in RKO and HCT-8 cells (n = 3; one-way ANOVA, *P* adjusted by FDR). **(C)** The colony formation assay of RKO and HCT-8 cells (n = 3; one-way ANOVA, *P* adjusted by FDR). **(D–G)** Wound healing, migration, and invasion assays were performed in RKO and HCT-8 cells (n = 3; one-way ANOVA, *P* adjusted by FDR). **(H, I)** Western blot showed altered protein levels of AKT-FOXO3/4 axis in empty vector, PIGR-overexpressing, LAMB3-overexpressing and rescue experiment group in RKO and HCT-8 cells. Differences compared to Vector group. Data are presented as the mean ± S.D. *P < 0.05; **P < 0.01; ***P < 0.001; ****P < 0.0001.

To better illustrate the role of PIGR in the regulation of LAMB3, we performed rescue assays in RKO and HCT-8 cells. The results showed that overexpression of LAMB3 promoted cell proliferation, migration and invasion *in vitro* and that re-expression of LAMB3 in PIGR-overexpressing cells was able to neutralize the antitumor effect of PIGR (**Figures 4A**–**G**).

### PIGR suppresses the AKT-FOXO3/4 axis by downregulating LAMB3

Laminin can interact with integrins to regulate multiple signaling pathways, such as the MAPK (25–27) and the AKT pathways (28–30). In this study, we wanted to investigate whether PIGR affect these pathways by downregulating LAMB3. Toward this direction, we detected AKT and phosphorylated AKT protein expression levels in PIGR-overexpressing cells and found that PIGR could reduce the levels of phosphorylated AKT in both RKO and HCT-8 cells. Given that forkhead box O3/4 (FOXO3/4) phosphorylated by AKT promotes the protein degradation of FOXO3/4, we further detected FOXO3/4 protein level and observed that FOXO3/4 was preserved by PIGR overexpression.

We also performed rescue experiments in PIGR-overexpressing cells and found that re-expression of LAMB3 in PIGR-overexpressing cells could reactivate the AKT pathway and inhibit FOXO3/4 by inducing protein degradation (**Figures 4H**, **I**).

### Tyrosine residue 743 of PIGR is essential for its antitumor effects

**Figure 5:**
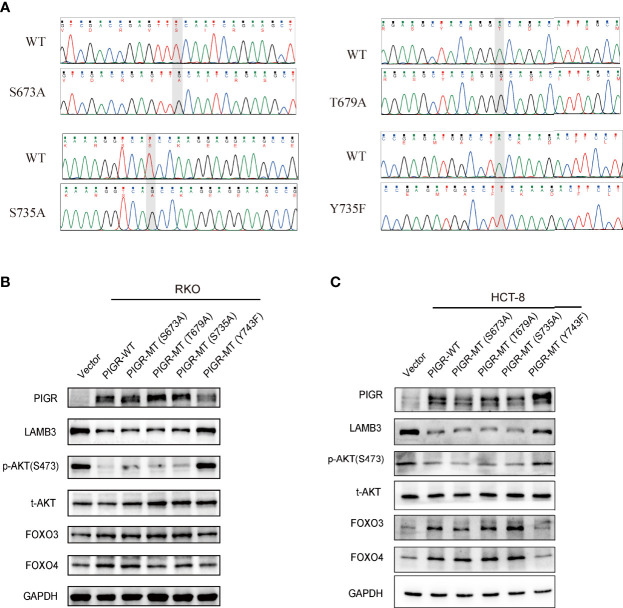
T743 of PIGR is essential for its anti-tumor effects **(A)** Successful point mutations of PIGR were confirmed by Sanger sequencing. **(B, C)** Western blot showed altered protein levels of AKT-FOXO3/4 axis in empty vector (Lane 1), PIGR wild-type (Lane 2), PIGR-S673A (Lane 3), PIGR-T679A (Lane 4), PIGR-S735A (Lane 5) and PIGR-Y743F (Lane 6) in RKO and HCT-8 cells.

A previous study reported that PIGR plays a signal transduction role by interacting with some transcription factors, e.g., Samd2/3 (18). Evidence indicates that phosphorylation of serine, threonine and tyrosine residues in the cytoplasmic tail of PIGR plays a role in signal transduction. Hence, to determine which amino residue of PIGR is necessary for its antitumor effects, we generated several point mutations in serine residues 673 (S673A) and 735 (S735A), threonine residue 679 (T679A), and tyrosine residue 743 (Y743F). The mutation plasmids were validated by Sanger sequencing (**Figure 5A**). Our data demonstrated that a mutation in tyrosine residue 743 (Y743F) weakened the effect of PIGR on downregulating LAMB3 and deactivating the AKT-FOXO3/4 signaling pathway in CRC cells. However, the S673A, S735A and T679A mutations had minimal effects on the antitumor ability of PIGR (**Figures 5B**, **C**).

### RE1 silencing transcription factor interacts with PIGR and transcriptionally inhibits LAMB3

**Figure 6:**
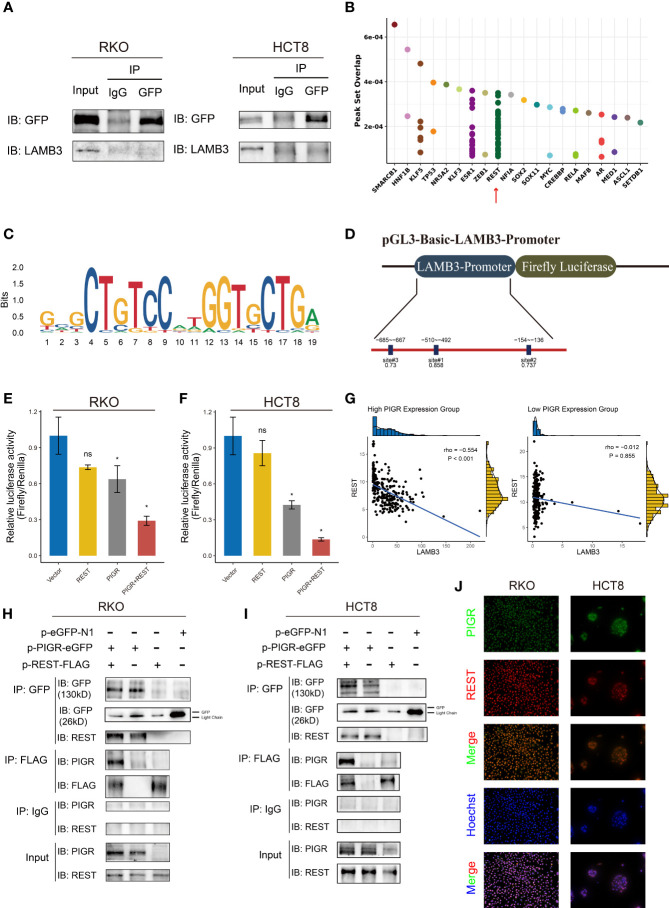
REST transcriptionally downregulated LAMB3 by interacting with PIGR **(A)** Co-immunoprecipitation assays detected the interaction between PIGR and LAMB3. IgG was used as control. **(B)** Predicted transcription factors bind to LAMB3 promoter using Cistrome database. **(C)** The binding motif of REST. **(D)** Schematic description of luciferase reporter and candidate REST binding sites in LAMB3 promoter. **(E, F)** Relative luciferase activities of LAMB3 wild-type promoter of empty vector (Vector), REST-overexpressing (REST), PIGR-overexpressing (PIGR) and co-expression of PIGR and REST (PIGR & REST) in RKO **(E)** and HCT-8 **(F)** cells. Differences compared with Vector group. **(G)** Correlation between expression of REST and LAMB3 in high PIGR expression group and low PIGR expression group. **(H, I)** Co-immunoprecipitation assays detected the interaction between PIGR and REST in RKO **(H)** and HCT-8 **(I)** cells. **(J)** The co-localization of PIGR and REST was confirmed by fluorescence microscopy. Data are presented as the mean ± S.D. ns, not significant; *P < 0.05.

Given the observed antitumor effect of PIGR through the downregulation LAMB3, we then explored the relationship between PIGR and LAMB3. To detect whether PIGR and LAMB3 interact, a coimmunoprecipitation assay was performed. However, the results did not support an interaction between PIGR and LAMB3 (**Figure 6A**).

To determine how PIGR downregulates LAMB3 expression, the promoter region of LMAB3 was analyzed. Using the Cistrome database (31), RE1 silencing transcription factor (REST) was recognized as a latent transcription factor of LAMB3 (**Figure 6B**). Then, we found several REST bindings sites that were predicted by the JASPAR database in LAMB3 promoter (**Figure 6C**) (32). Moreover, an interesting result was observed that REST and LAMB3 expression exhibited a stronger negative correlation in the group with relatively high PIGR expression (r = -0. 554) compared with the low PIGR expression group (r = -0.012) (**Figure 6G**). Thus, we assumed that PIGR might interact with REST and enhance the transcriptional inhibition effect of REST in LAMB3 promoter.

We performed a dual luciferase reporter assay to test whether REST could transcriptionally inhibit LAMB3 expression (**Figure 6D**). The results revealed that REST could attenuate the luciferase activity generated by the LAMB3 promoter. Furthermore, REST presented stronger inhibition of LAMB3 in the presence of PIGR (**Figures 6E**, **F**). To perform a coimmunoprecipitation assay, RKO and HCT-8 cells were transfected with plasmids containing PIGR tagged with GFP and/or REST tagged with Flag. Our data showed that the protein complex precipitated by the GFP antibody contained REST, and the protein complex precipitated by Flag antibody also contained PIGR (**Figures 6H**, **I**). Likewise, immunofluorescence assay showed the co-localization of PIGR and REST (**Figure 6J**). Taken together, our results indicated that REST interacts with PIGR and transcriptionally inhibits LAMB3 expression.

### Cells overexpressing PIGR are more sensitive to chemotherapy drugs

**Figure 7:**
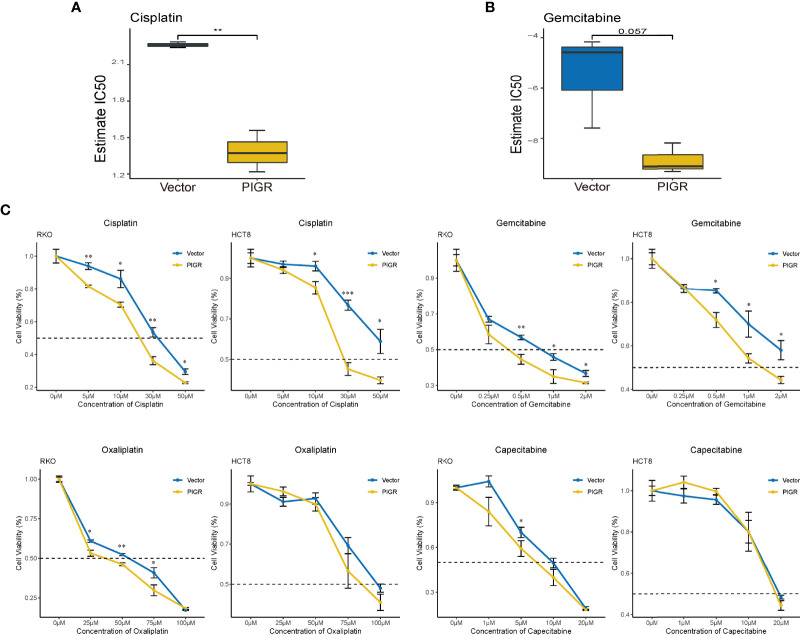
PIGR overexpression increases chemotherapy sensitivity in CRC cells **(A, B)** Estimated IC50 of cisplatin **(A)** and gemcitabine **(B)** in RKO and PIGR overexpression RKO cells. **(C)** Cell viability after treated with a series concentration of cisplatin, gemcitabine, oxaliplatin and capecitabine of RKO and HCT-8 cells. *P < 0.05; **P < 0.01; ***P < 0.001.

Finally, we tested whether PIGR expression is associated with sensitivity to certain chemotherapy drugs in RKO and HCT-8 cells. The estimated IC_50_ values of drugs or chemicals using our RNA-seq data were calculated with the “pRRophetic” package (**Table S6**). Cisplatin and gemcitabine were identified because PIGR-overexpressing cells had lower IC_50_ values for these drugs compared with control cells (**Figures 7A**, **B**). To further validate the bioinformatics analysis, a CCK-8 assay was performed to detect CRC cell sensitivity to cisplatin, gemcitabine and two analog drugs, oxaliplatin and capecitabine, which are the clinical first-line drugs for patients with CRC. Our data suggested that PIGR-overexpressing cells were more sensitive to cisplatin and gemcitabine but not to oxaliplatin and capecitabine (**Figure 7C**).

## Discussion

PIGR is widely expressed in the mucosal epithelium and can be upregulated by inflammatory cytokines during viral or bacterial infection (33, 34). Previous studies have shown that PIGR is upregulated in several malignancies, such as hepatocellular carcinoma (18) and osteosarcoma (13), and is downregulated in other cancers, including pancreatic cancer (14), breast cancer (15), ovarian cancer (16), and stomach and esophageal cancer (17). Mechanistically, PIGR promotes hepatocellular carcinoma by activating the Smad and Yes-MEK/ERK signaling pathways (18, 19). On the other hand, PIGR inhibits lung cancer by downregulating NOTCH3 (21). However, dysregulation of PIGR in CRC remain unknown.

Our study showed that the differences between CRC and adjacent normal tissues in methylation, mRNA and protein expression were significant and that PIGR methylation and expression varied with the tumor stage. Kaplan–Meier curve and univariate Cox regression analysis indicated that both PIGR methylation and expression levels were prognostic markers of CRC patients. However, multivariate Cox regression showed that PIGR was not an independent prognostic marker potentially because PIGR exhibits strong collinearity with tumor stage in the multivariate regression model. Our results were further validated by a public database with a large sample size. These data showed that PIGR might be involved in inhibiting the tumorigenesis of CRC.

For demethylation assay, cells were treated with a series of 5-Aza concentrations (0-12.5 μM). In our data, we observed that the expression in the 12.5 μM group decreased significantly. We hypothesized that the cytotoxicity caused by excessive 5-Aza led to cell death. In summary, the expression of PIGR mRNA is inversely regulated by PIGR methylation in CRC cells.

Our data showed that PIGR played an antitumor role in CRC, including the inhibition of CRC cell proliferation and migration. To explore the downstream target of PIGR, we performed RNA-seq in PIGR-overexpressing cells and control cells. The ECM-receptor interaction pathway was identified to be the most downregulated pathway by PIGR. The ECM is involved in the formation of the microenvironment. ECM components dysfunction and remodeling are attributed to tumor progression in diverse tumors (35–37). We considered LAMB3 as a downstream target of PIGR because it was among the core genes in the ECM-receptor pathway identified by GSEA. In addition, LAMB3 had the largest absolute difference in TPM between PIGR-overexpressing cells and control cells. Then, LAMB3 and PIGR expression was further validated by RT–qPCR and western blot.

LAMB3, a subunit of laminin-332, promotes tumor progression, as reported by previous studies (38). Laminin-332 regulates downstream signal transduction by interacting with integrins on the cell surface (39). Evidence has shown that the interaction of laminin-332 with integrins activates several cancer-related signaling pathways such as PI3K/AKT (40), MAPK/ERK (41), JNK and NF-κB (42). Recent studies described that upregulated LAMB3 activated AKT in pancreatic cancer (30), thyroid cancer (43) and CRC (28). Here, we assumed that PIGR suppresses the activation of the AKT pathway by downregulating LAMB3. Our data showed that AKT phosphorylation levels in PIGR-overexpressing cells was significantly lower than that of control cells, whereas AKT phosphorylation levels were rescued when LAMB3 was re-expressed in PIGR-overexpressing cells. In addition, proliferation and migration were also recovered in re-expressed LAMB3 cells. An inverse correlation between phosphorylated AKT and FOXO3/4 was observed given that AKT activated by LAMB3 can induce degradation of FOXO3/4 (44). Taken together, we accept the above assumption that PIGR suppresses the AKT-FOXO3/4 axis by downregulating LAMB3.

PIGR is a transmembrane protein whose extracellular domain binds to dIgA and pIgM, while the transcytosis needs the cytoplasmic tail of PIGR (10, 11). A previous study indicated that mutations in serine residues (S682A and S734A) of PIGR are crucial for PIGR-induced EMT (18). However, our data demonstrated that the mutation in tyrosine residue (Y743F) of PIGR is important to its antitumor effects. Tyrosine 743 of PIGR is considered to play a critical role in transcytosis during the immune response (45). Therefore, we believe that PIGR regulates downstream target genes and pathways in the process of transcytosis. When transcytosis is lost due to a mutation in tyrosine residues 743, the signal transduction ability of PIGR is also abolished.

To further understand the relationship between PIGR and LAMB3, we performed a coimmunoprecipitation assay to detect whether PIGR directly interacts with LAMB3. Unfortunately, PIGR does not interact with LAMB3. A previous study reported that PIGR could interact with certain transcription factors. Thus, we predicted transcriptional regulators using website tools. Several transcriptional regulators had been identified by the website tools (**Figure 6B**). Considering PIGR inversely correlated with LAMB3 and no interaction between them, we tented to seek transcription repressors. Besides, the research evidence of REST enriches in LAMB3 promoter are sufficient. As we saw in the **Figure 6B**, dots indicated the number of studies that detected REST enrichment to the LAMB3 promoter by CHIP-Seq. Eventually, REST was recognized to be the regulator of LAMB3. REST is highly expressed in nonneural cells and participates in cell differentiation and proliferation (46). Previous studies found that REST could regulate the ECM and that REST-mediated regulation of ECM was crucial for neural development (47, 48). Thus, a dual luciferase reporter assay was performed to detect the regulation between REST and LAMB3. A relationship in which REST transcriptionally inhibited LAMB3 was identified. Moreover, in the presence of PIGR, REST could inhibit LAMB3 transcription. However, in the absence of PIGR, the inhibition almost disappeared. Interestingly, the same trend was found in normal colon tissues based on GTEx data. In high PIGR-expressing group, REST had a strong negative correlation with LAMB3. In contrast, in low PIGR-expressing group, REST exhibited no correlation with LAMB3. Then, coimmunoprecipitation and immunofluorescence assay were performed and the results showed that PIGR physically interacts with REST.

Cells with high PIGR expression were more sensitive to cisplatin and gemcitabine predicted by algorithm. Then, drug sensitivity was confirmed using CCK-8 assays. Recently, a study showed that siRNA-mediated silencing of LAMB3 could enhance the sensitivity of head and neck squamous cell carcinoma cells to cisplatin (49), which may explain why PIGR can also increase the sensitivity of CRC cells to cisplatin. These results provide new clues for precision treatment of CRC patients, but further verification is needed.

In summary, we demonstrated that both PIGR methylation and expression are prognostic markers of CRC patients. In addition, the PIGR-REST-LAMB3-AKT/FOXO3/4 axis was recognized to be the underlying mechanism by which PIGR inhibits CRC tumorigenesis. At last, PIGR-overexpressing cells were more sensitive to cisplatin and gemcitabine, which provides clues for CRC treatment.

## Conclusions

Methylation and expression of PIGR serve as a powerful prognostic biomarker for CRC patients. PIGR suppresses colorectal cancer through the AKT-FOXO3/4 axis by downregulating LAMB3 expression, which may provide a clue for molecular treatment for CRC.

## Data availability statement

The datasets presented in this study can be found in online repositories. The names of the repository/repositories or accession number(s) can be found in the Material and method section.

## Ethics statement

The studies involving human participants were reviewed and approved by the ethics committee in Harbin Medical University. The patients/participants provided their written informed consent to participate in this study. The animal study was reviewed and approved by the Animal Research Ethics Committee of the Harbin Medical University.

## Author contributions

YZ initiated, designed, and coordinated the study. DZ, HH, TZ, LZ, BC, YL, ST, LYZ, LG, QF, and ZC performed tissue sample collection, DNA, RNA and protein extraction. DZ and TZ performed cell biology and molecular biology experiments. DZ, HH, and TT performed data analysis and interpretation. DZ wrote the manuscript. YZ and DZ revised the manuscript. All authors contributed to the article and approved the submitted version.

## Funding

This work was funded by the National Natural Science Foundation of China (grant number 82073643) and the special fund for the construction of high-level universities and advantageous and characteristic disciplines in Heilongjiang province.

## Acknowledgments

We sincerely thank all the patients who participated the studies from TCGA and GEO datasets and this study and the experimental animals sacrificed for this study.

## Conflict of interest

The authors declare that the research was conducted in the absence of any commercial or financial relationships that could be construed as a potential conflict of interest.

## Publisher’s note

All claims expressed in this article are solely those of the authors and do not necessarily represent those of their affiliated organizations, or those of the publisher, the editors and the reviewers. Any product that may be evaluated in this article, or claim that may be made by its manufacturer, is not guaranteed or endorsed by the publisher.
