# Corrigendum: Polymeric immunoglobulin receptor suppresses colorectal cancer through the AKT-FOXO3/4 axis by downregulating LAMB3 expression

**DOI:** 10.3389/fonc.2022.1012871

**Published:** 2022-08-22

**Authors:** Ding Zhang, Hao Huang, Ting Zheng, Lei Zhang, Binbin Cui, Yanlong Liu, Shiheng Tan, Liyuan Zhao, Tian Tian, Lijing Gao, Qingzhen Fu, Zesong Cheng, Yashuang Zhao

**Affiliations:** ^1^ Department of Epidemiology, School of Public Health, NHC Key Laboratory of Etiology and Epidemiology (23618504), Harbin Medical University, Harbin, China; ^2^ Department of Colorectal Surgery, The Third Affiliated Hospital of Harbin Medical University, Harbin, China

**Keywords:** colorectal cancer, polymeric immunoglobulin receptor, prognostic marker, AKTFOXO3/4 axis, methylation-driven gene

## Error in Figure/Table

In the published article, there was an error in **Figure 4** as published. The microscopy slides of migration of “Vector” group and “PIGR+ LAMB3” group were misused in **Figure 4G** due to carelessness. The corrected **Figure 4** and its caption appear below.

**Figure 4:**
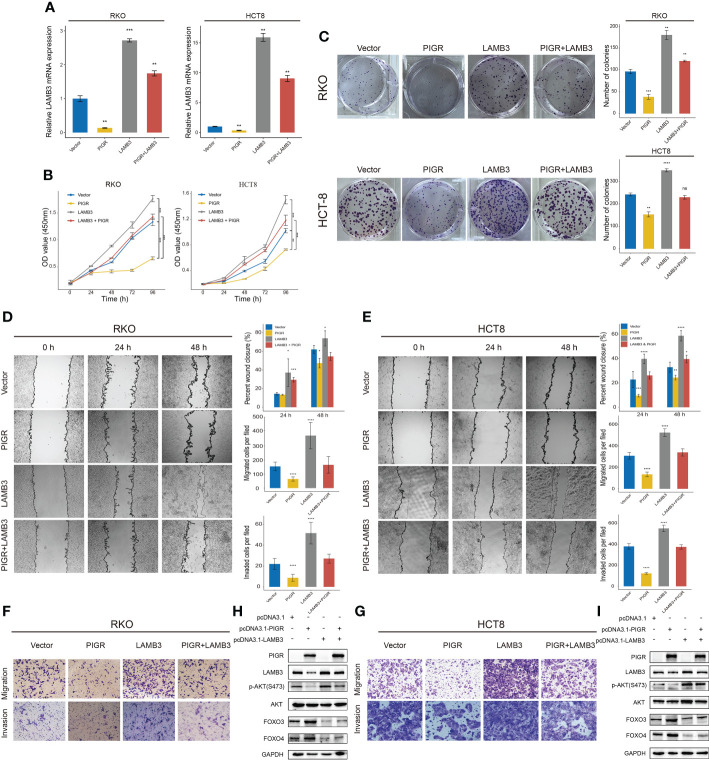
PIGR suppresses AKT-FOXO3/4 axis through LAMB3 **(A)** Relative LAMB3 mRNA expression of empty vector (Vector), PIGR-overexpressing (PIGR), LAMB3-overexpressing (LAMB3) and rescue experiment group (PIGR & LAMB3) in RKO and HCT-8 cells. Differences compared to Vector group (n = 3; one-way ANOVA, P adjusted by FDR). **(B)** Cell proliferation was detected by CCK-8 assay at 0, 24, 48, 72 and 96 hours in RKO and HCT-8 cells (n = 3; one-way ANOVA, P adjusted by FDR). **(C)** The colony formation assay of RKO and HCT-8 cells (n = 3; one-way ANOVA, P adjusted by FDR). **(D–G)** Wound healing, migration, and invasion assays were performed in RKO and HCT-8 cells (n = 3; one-way ANOVA, P adjusted by FDR). **(H, I)** Western blot showed altered protein levels of AKT-FOXO3/4 axis in empty vector, PIGR-overexpressing, LAMB3- overexpressing and rescue experiment group in RKO and HCT-8 cells. Differences compared to Vector group. Data are presented as the mean ± S.D. *P < 0.05; **P < 0.01; ***P < 0.001; ****P < 0.0001.

The authors apologize for this error and state that this does not change the scientific conclusions of the article in any way. The original article has been updated.

## Publisher’s note

All claims expressed in this article are solely those of the authors and do not necessarily represent those of their affiliated organizations, or those of the publisher, the editors and the reviewers. Any product that may be evaluated in this article, or claim that may be made by its manufacturer, is not guaranteed or endorsed by the publisher.

