# Supplementary material for: Polymeric immunoglobulin receptor suppresses colorectal cancer through the AKT-FOXO3/4 axis by downregulating LAMB3 expression
Source: Front Oncol. 2022 Aug 5;12:924988. doi: 10.3389/fonc.2022.924988 (PMC9389318; doi:10.3389/fonc.2022.924988)
Supplement: Supplementary file 1 [file DataSheet_1.docx]

Supplementary Material

# Supplementary Methods

## Plasmid construction

The full-length cDNA coding sequence of PIGR was generated by reverse transcription-polymerase chain reaction (RT-PCR). PCR was performed using a high-fidelity polymerase kit (KOD -Plus- NEO, Toyobo, Japan) and PCR products were purified using a gel extraction kit (E.Z.N.A.® MicroElute Gel Extraction Kit, Omega Biotek) following the recommended protocols in the manufacturer’s instructions. Then PCR products and the mammalian expression vector pCDNA3.1(-) were digested by EcoRI and HindIII (NEB) at 37 ℃ for 2 h and the cohesive end was ligated by T4 DNA ligase (NEB) at 16 ℃ overnight. The recombinant plasmid pCDNA3.1-PIGR was transformed into E. coli DH5α. Positive clones were screened and confirmed by double restriction enzyme digestion, and the insert fragment was sequenced by Sanger sequencing (Sangon Biotech). The DH5α strain containing the pCDNA3.1-PIGR recombinant plasmid was cultured in LB medium with 100 μg/ml ampicillin, and the endotoxin-free plasmid was obtained with a commercial kit (Omega Biotek) and stored at -20 ℃ before use.

The construction steps of pCDNA3.1-LAMB3, pCMV-REST-3×Flag and pEGFP-N1-PIGR were the same as note above. Specifically, the restriction endonuclease pairs were XhoI and EcoRI, HindIII and XhoI and EcoRI and XmaI. The stop codons of REST and PIGR in pCMV-REST-3×Flag and pEGFP-N1-PIGR were removed to merge Flag or GFP.

The mutation plasmid of pCDNA3.1-PIGR was generated by using the DpnI digestion method. In brief, the mutation plasmids were amplified by PCR using pCDNA3.1-PIGR as template and a pair of reverse complementary primers that contained mutation sites. Then, the original templates were digested by DpnI. Eventually, the nicked mutation plasmids were transformed into E. coli DH5α.

For the luciferase reporter assay, reporter plasmids were constructed by the homologous recombination method. In short, the LAMB3 promoter was amplified with homologous arms and ligated to the linearized pGL3-basic vector with a 20-bp overlap using the Seamless Cloning Kit (Beyotime).

The primers used for gene amplification are shown in Table S7.

## Western blot and immunoprecipitation

For western blot, cells were lyses in RIPA (strong) buffer (Beyotime) supplemented with 1% (v/v) 100 mM PMSF and phosphatase inhibitors (Roche). Lysates were clarified by supersonic, then, the supernatant was collected after centrifuged for 10 minutes. The total protein concentration is quantified by BCA methods. Protein samples were subjected to SDS-PAGE and transferred onto PVDF membranes using Tanon transfer apparatus. Membranes were blocked with 5% non-fat milk or BSA in TBS containing 0.1% Tween-20 (TBST) at room temperature for 1-4 hours (depend on different antibodies), followed by incubation with primary antibody against PIGR (Genetex, GTX110197, 1:3000), LAMB3 (Affinity Bioscience, DF2381, 1:2000), REST (Bimake, A5475, 1:2000), total AKT (Bimake, A5031, 1:2000), Phospho-AKT-ser473 (Bimake, A5030, 1:2000), GFP (Proteintech, 50430-2-AP, 1:4000), Flag (Proteintech, 80010-1-RR, 1:3000), GAPDH (Affinity Bioscience, AF7021, 1:10000) overnight at 4°C. Membranes were washed 3 × 10 minutes in TBST and incubated with species-specific HRP-conjugated secondary antibodies (Jacksonimmuno) for 1 hour at room temperature. After 3 × 10 minutes washes in TBST, membranes were covered by an enhanced chemiluminescence (ECL) reagent before imaged by chemiluminescent imaging system (Tanon).

For immunoprecipitation, cells were lyses in RIPA (weak, non-ionic detergent) buffer (Beyotime) supplemented with 1% (v/v) 100 mM PMSF and phosphatase inhibitors (Roche). Lysates were clarified by supersonic, then, the supernatant was collected after centrifuged for 10 minutes. Lysates were then incubated with IP antibody overnight at 4°C. For Flag and GFP IP, 2 μg of anti-Flag antibody or anti-GFP antibody (Proteintech) was used for 2000 ug of total protein lysate. Subsequently, the antigen-antibody complex was incubated with protein A/G magnet beads (Bimake) for 2 hours at 4°C. Last, beads were washed by IP lysis buffer, boiled in 1× SDS gel loading buffer, and subjected to electrophoresis as described above.

## RNA-seq

RKO cells which stably transfected with pCDNA3.1-PIGR and pCDNA3.1(-) vector were collected for RNA sequencing. Total RNA was extracted using TRIzol, following by the manufacturer’s instructions. There were three replicates for both groups. The quality and concentration of total RNA were examined by agarose gel electrophoresis and a spectrophotometer. cDNA libraries were synthesized and sequenced by Illunima HiSeq 2000 platform by the Bioacme Biotechnology Co., Ltd. (Wuhan, China). The clean data were mapped to Homo sapiens GRCh38 using Hisat2 with default settings. Gene expression level were normalized by Transcripts Per Million (TPM) method.

# Supplementary Figures and Tables

## Supplementary Figures


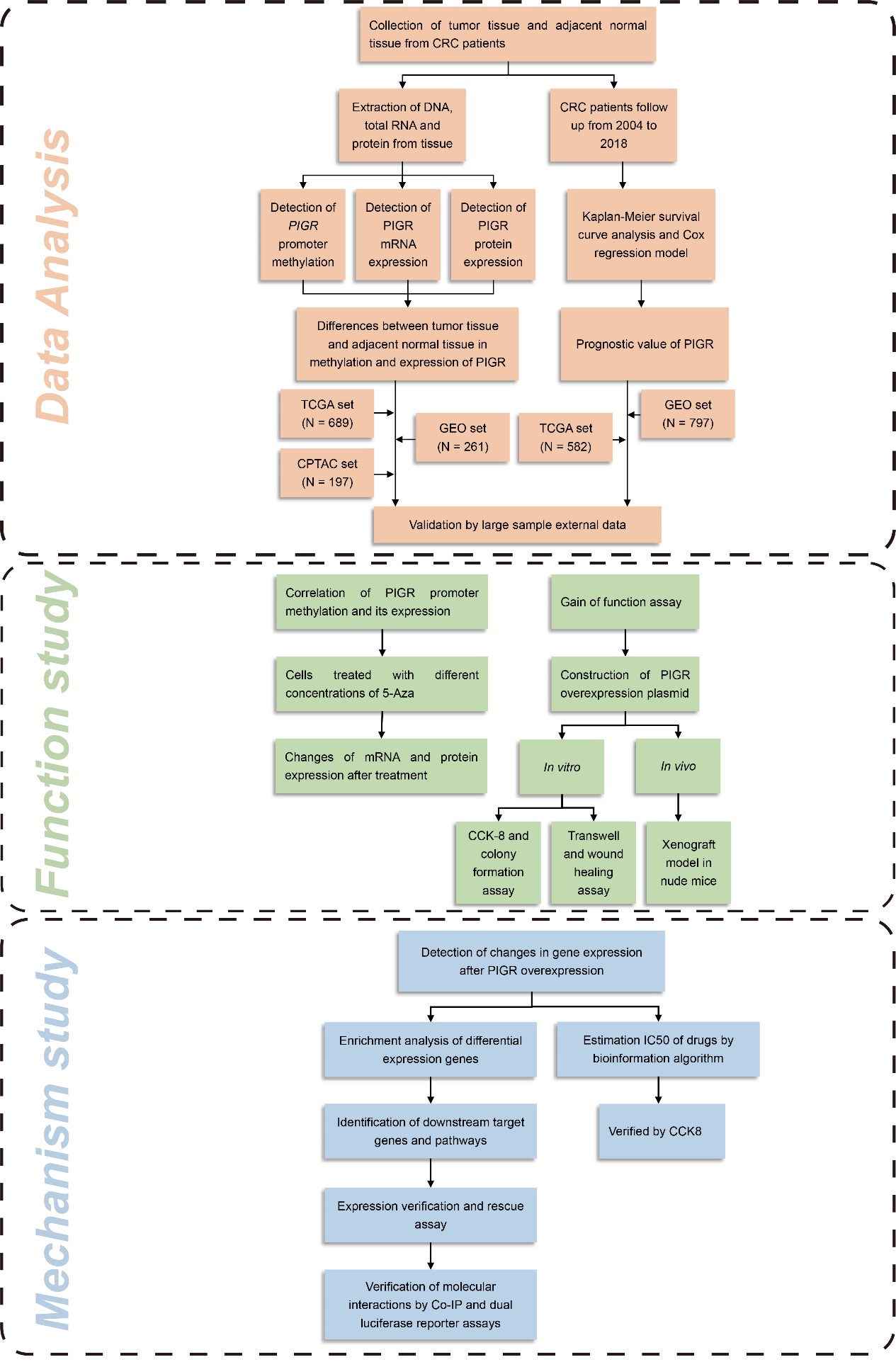


**Supplementary Figure 1.** A workflow of the present study.


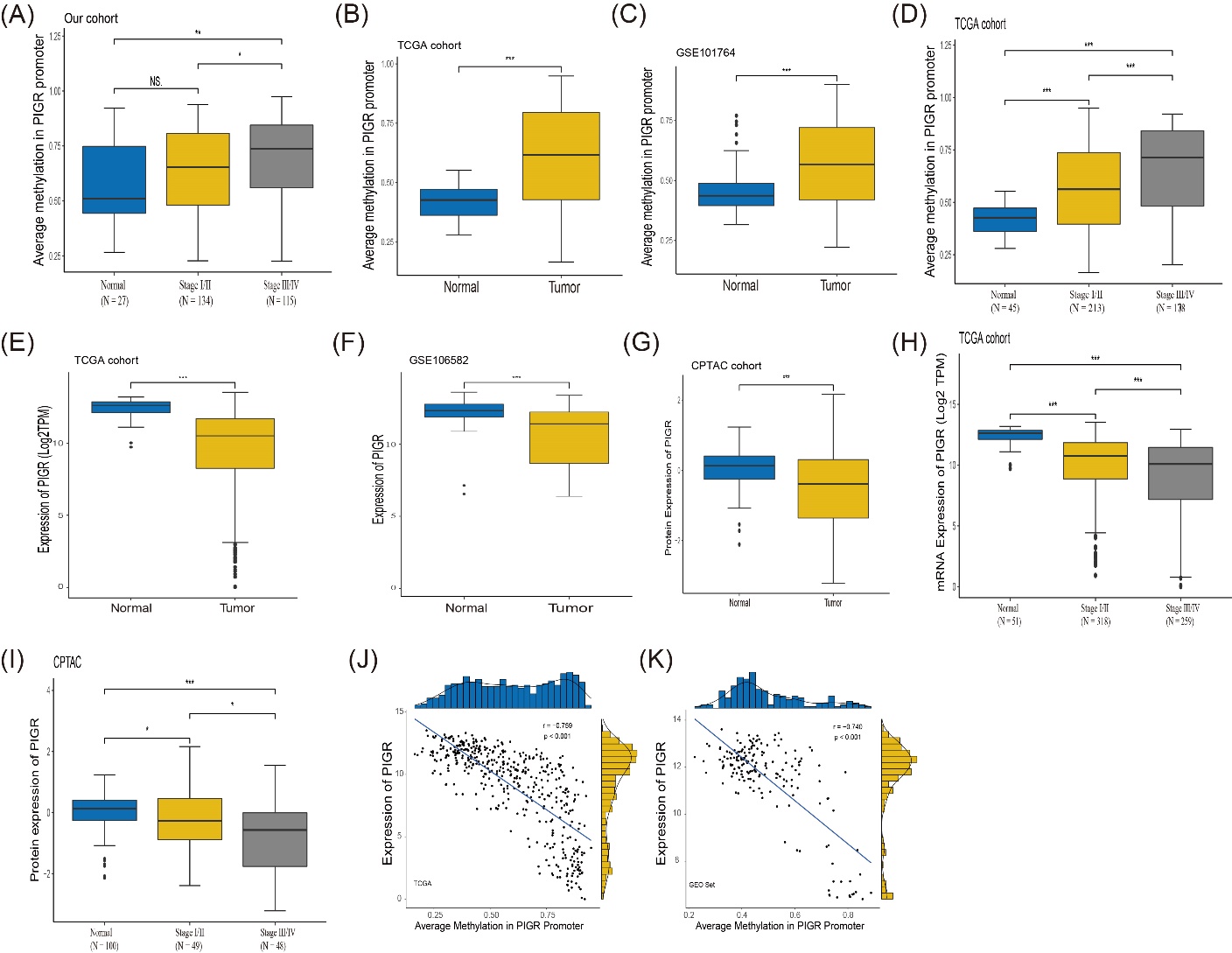


**Supplementary Figure 2.** PIGR is hypermethylated and downregulated in CRC tissues. (A) Methylation in PIGR promoter is increasing with the increased tumor stage in our cohort. (B-C) The methylation of PIGR promoter in adjacent normal tissues (N = 45 and N = 149) and CRC tissues (N = 414 and N = 112) in TCGA and GEO. (D) Methylation in PIGR promoter is increasing with the increased tumor stage in TCGA. (E-F) The PIGR mRNA expression in CRC tissues (N = 51 and N = 108) and adjacent normal tissues (N = 638 and N = 77) in TCGA and GEO. (G) Quantify PIGR protein expression in CRC tissues (N = 97) and normal tissues (N =100) of CPTAC database. (H-I) mRNA and protein expression of PIGR is decreasing with the increased tumor stage in TCGA cohort (H, mRNA expression) and CPTAC (I, protein expression). (J-K) Correlation between methylation level of PIGR promoter and its mRNA expression in TCGA and GEO cohorts.


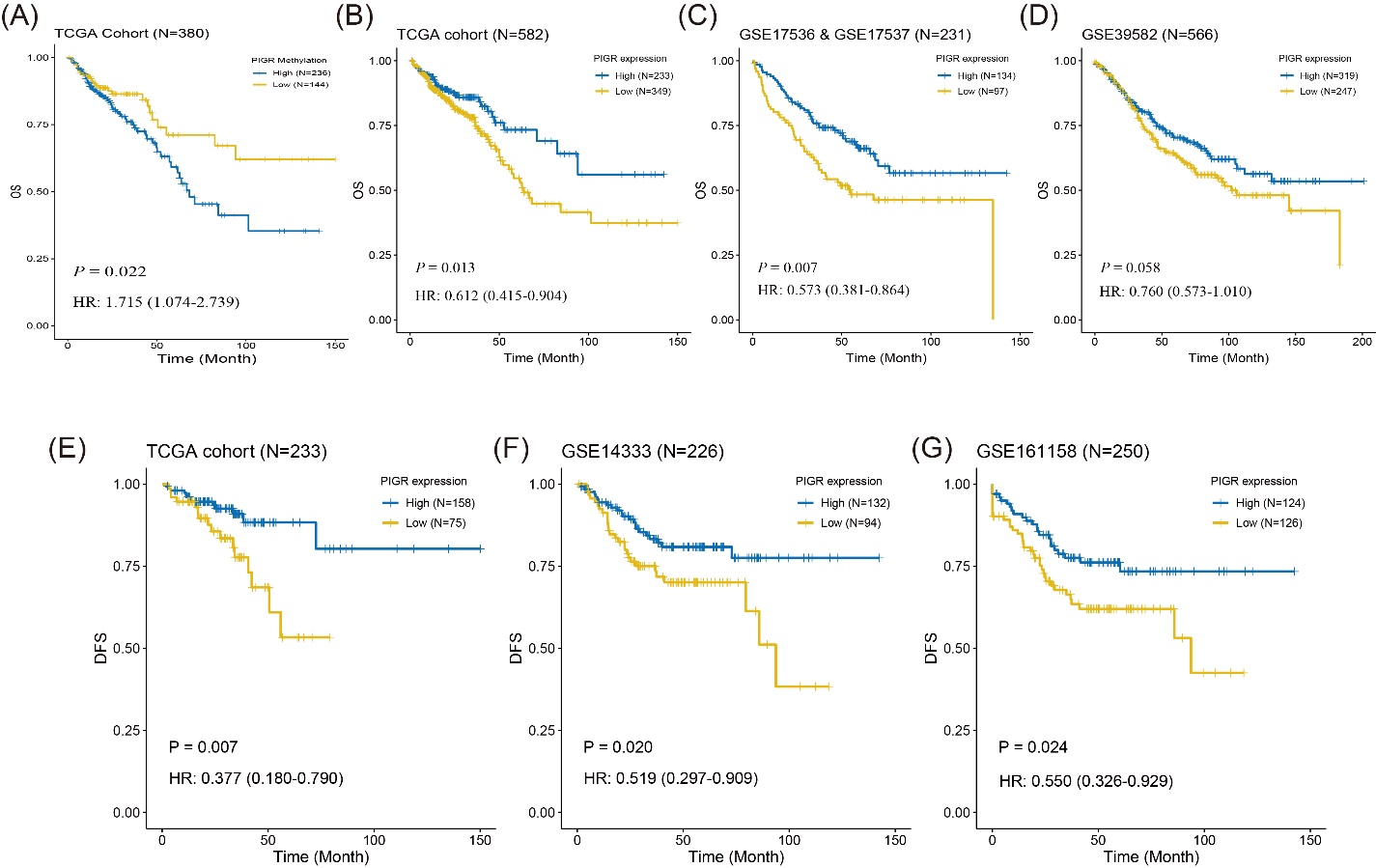


**Supplementary Figure 3.** Hypermethylated and downregulated PIGR is correlated with poor prognosis. (A) Kaplan-Meier curve of OS based on the methylation of PIGR promoter in tumor tissues of TCGA cohort. (B-D) Kaplan-Meier curve of OS based on the PIGR expression in tumor tissues of TCGA and GEO cohorts. (E-G) Kaplan-Meier survival analysis of disease-free survival based on PIGR expression in the tumor tissues from TCGA cohort and GEO datasets


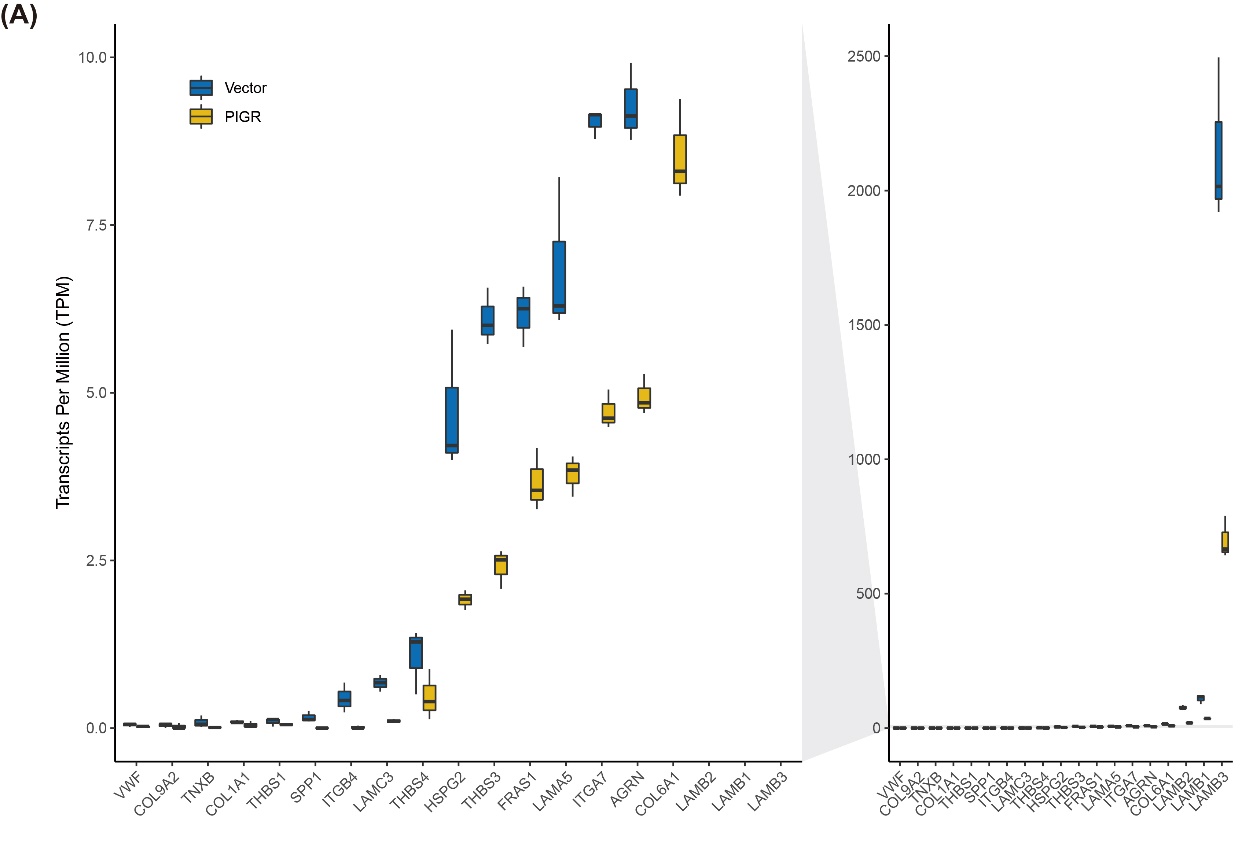


**Supplementary Figure 4.** Difference of Core genes in ECM-Interaction Receptor pathway. (A) Expression of 12 core genes in ECM-interaction receptor pathway between empty vector group and PIGR overexpression group.

## Supplementary Tables

Table S1. Association between *PIGR* methylation and overall survival of CRC Patients in our cohort.

| **Variable** | **Univariate Analysis** | |  | **Multivariate Analysis**^a^ | |
| --- | --- | --- | --- | --- | --- |
|  | HR (95% CI) | *P* Value |  | HR (95% CI) | *P* Value |
| Our Cohort (N = 248) |  |  |  |  |  |
| **PIGR Methylation** (Hyper- versus Hypo-) | 1.606 (1.109-2.325) | **0.012** |  | 1.423 (0.98-2.067) | 0.064 |
| **Sex**  (Male versus Female) | 0.906 (0.628-1.307) | 0.597 |  | 0.925 (0.641-1.335) | 0.676 |
| **Age**  (≥65 versus <65) | 1.284 (0.891-1.850) | 0.180 |  | 1.440 (0.995-2.084) | 0.053 |
| **Stage**  (III or IV versus I or II) | 2.312 (1.597-3.347) | **<0.001** |  | 2.334 (1.601-3.404) | **<0.001** |

Abbreviations: CRC, colorectal cancer; CI, confidence interval; Hyper-, hypermethylation; Hypo-, hypomethylation; HR, hazard ratio.

^a^Multivariate adjusted factors: age, sex and tumor stage.

Table S2. Association between *PIGR* methylation and overall survival of CRC Patients in TCGA.

| **Variable** | **Univariate Analysis** | |  | **Multivariate Analysis**^a^ | |
| --- | --- | --- | --- | --- | --- |
|  | HR (95% CI) | *P* Value |  | HR (95% CI) | *P* Value |
| TCGA Cohort (N = 361) |  |  |  |  |  |
| **PIGR Methylation** (Hyper- versus Hypo-) | 1.715 (1.074-2.739) | **0.024** |  | 1.524 (0.922-2.521) | 0.101 |
| **Sex**  (Male versus Female) | 1.360 (0.883-2.096) | 0.163 |  | 1.094 (0.688-1.739) | 0.704 |
| **Age**  (≥65 versus <65) | 2.005 (1.258-3.195) | **0.003** |  | 2.364 (1.429-3.913) | **0.001** |
| **Stage**  (III or IV versus I or II) | 2.688 (1.681-4.296) | **<0.001** |  | 2.842 (1.763-4.580) | **<0.001** |

Abbreviations: CRC, colorectal cancer; CI, confidence interval; Hyper-, hypermethylation; Hypo-, hypomethylation; HR, hazard ratio.

^a^Multivariate adjusted factors: age, sex and tumor stage.

**Table S3**. Association between PIGR expression and overall survival of CRC Patients in TCGA.

| **Variable** | **Univariate Analysis** | |  | **Multivariate Analysis**^a^ | |
| --- | --- | --- | --- | --- | --- |
|  | HR (95% CI) | *P* Value |  | HR (95% CI) | *P* Value |
| TCGA cohort (N = 562) |  |  |  |  |  |
| **PIGR Expression**  (High versus Low) | 0.612 (0.415-0.904) | **0.014** |  | 0.683 (0.455-1.026) | 0.067 |
| **Sex**  (Male versus Female) | 1.102 (0.769-1.580) | 0.595 |  | 0.958 (0.657-1.397) | 0.823 |
| **Age**  (≥65 versus <65) | 1.829 (1.226-2.730) | **0.003** |  | 2.340 (1.525-3.592) | **<0.001** |
| **Stage**  (III or IV versus I or II) | 3.324 (2.228-4.960) | **<0.001** |  | 3.489 (2.325-5.236) | **<0.001** |

Abbreviations: CRC, colorectal cancer; CI, confidence interval; HR, hazard ratio.

^a^Multivariate adjusted factors: age, sex and tumor stage.

**Table S4**. Association between PIGR expression and overall survival of CRC Patients in GSE17536 & 17537.

| **Variable** | **Univariate Analysis** | |  | **Multivariate Analysis**^a^ | |
| --- | --- | --- | --- | --- | --- |
|  | HR (95% CI) | *P* Value |  | HR (95% CI) | *P* Value |
| GEO cohort (N = 231) |  |  |  |  |  |
| **PIGR Expression**  (High versus Low) | 0.573 (0.381-0.864) | **0.008** |  | 0.660 (0.437-0.996) | **0.048** |
| **Sex**  (Male versus Female) | 0.995 (0.659-1.501) | 0.98 |  | 1.043 (0.685-1.588) | 0.844 |
| **Age**  (≥65 versus <65) | 1.050 (0.697-1.581) | 0.817 |  | 1.398 (0.912-2.143) | 0.124 |
| **Stage**  (III or IV versus I or II) | 3.865 (2.306-6.478) | **<0.001** |  | 3.993 (2.353-6.775) | **<0.001** |

Abbreviations: CRC, colorectal cancer; CI, confidence interval; HR, hazard ratio.

^a^Multivariate adjusted factors: age, sex and tumor stage.

**Table S5.** Association between PIGR expression and overall survival of CRC Patients in GSE39582.

| **Variable** | **Univariate Analysis** | |  | **Multivariate Analysis**^a^ | |
| --- | --- | --- | --- | --- | --- |
|  | HR (95% CI) | *P* Value |  | HR (95% CI) | *P* Value |
| GEO cohort (N = 561) |  |  |  |  |  |
| **PIGR Expression**  (High versus Low) | 0.760 (0.573-1.010) | 0.059 |  | 0.864 (0.647-1.155) | 0.324 |
| **Sex**  (Male versus Female) | 1.31 (0.980-1.750) | 0.068 |  | 1.415 (1.057-1.893) | **0.020** |
| **Age**  (≥65 versus <65) | 1.472 (1.082-2.002) | **0.014** |  | 1.596 (1.170-2.177) | **0.003** |
| **Stage**  (III or IV versus I or II) | 1.767 (1.326-2.354) | **<0.001** |  | 1.83 (1.363-2.456) | **<0.001** |

Abbreviations: CRC, colorectal cancer; CI, confidence interval; HR, hazard ratio.

^a^Multivariate adjusted factors: age, sex and tumor stage.

**Table S6**. Estimated IC_50_ in PIGR-overexpressing cells and control cells.

| **Drug** | **PIGR1** | **PIGR2** | **PIGR3** | **Vector1** | **Vector2** | **Vector3** |
| --- | --- | --- | --- | --- | --- | --- |
| A-443654 | -3.201 | -3.223 | -3.213 | -3.129 | -3.136 | -3.178 |
| A-770041 | 0.348 | -0.110 | 0.225 | 0.658 | 0.675 | 0.261 |
| ABT-263 | 4.421 | 4.428 | 4.232 | 4.311 | 4.283 | 3.826 |
| ABT-888 | 5.810 | 5.824 | 5.783 | 5.785 | 5.778 | 5.685 |
| AG-014699 | 5.845 | 5.698 | 6.314 | 5.709 | 5.800 | 6.461 |
| AICAR | 24.181 | 25.285 | 25.305 | 20.084 | 19.970 | 19.496 |
| AKT-inhibitor-VIII | 1.869 | 1.799 | 2.162 | 1.519 | 1.554 | 2.086 |
| AP-24534 | 3.815 | 3.967 | 3.718 | 2.289 | 2.205 | 1.809 |
| AS601245 | 0.023 | 0.017 | 0.037 | 0.039 | 0.035 | 0.055 |
| ATRA | 6.815 | 7.072 | 6.749 | 6.127 | 6.068 | 5.277 |
| AUY922 | -5.503 | -5.525 | -5.482 | -5.210 | -5.207 | -5.127 |
| Axitinib | 6.011 | 6.114 | 5.947 | 5.316 | 5.276 | 4.949 |
| AZD-0530 | 5.915 | 4.764 | 5.528 | 7.737 | 7.884 | 10.363 |
| AZD-2281 | 7.211 | 7.210 | 7.279 | 6.683 | 6.720 | 6.772 |
| AZD6244 | -1.864 | -1.732 | -1.719 | -1.605 | -1.626 | -1.735 |
| AZD6482 | 5.138 | 5.381 | 5.260 | 3.724 | 3.543 | 2.641 |
| AZD7762 | 16.442 | 17.688 | 16.717 | 8.603 | 8.480 | 6.626 |
| AZD8055 | 7.231 | 7.698 | 7.255 | 4.318 | 4.337 | 3.082 |
| BAY-61-3606 | 2.165 | 2.146 | 2.375 | 2.190 | 2.168 | 2.334 |
| Bexarotene | 1.343 | 1.285 | 1.528 | 0.405 | 0.409 | 0.306 |
| BI-2536 | -5.183 | -5.182 | -5.171 | -5.148 | -5.150 | -5.164 |
| BIBW2992 | 2.380 | 2.492 | 2.205 | 2.762 | 2.677 | 2.535 |
| Bicalutamide | 4.585 | 4.509 | 4.709 | 4.746 | 4.752 | 5.117 |
| BI-D1870 | 3.407 | 3.418 | 3.240 | 3.213 | 3.178 | 2.728 |
| BIRB-0796 | 5.470 | 5.597 | 5.413 | 5.986 | 5.903 | 5.856 |
| BMS-509744 | 0.772 | 0.777 | 0.899 | 0.909 | 0.872 | 0.914 |
| BMS-536924 | 2.739 | 3.067 | 3.081 | 0.180 | 0.092 | 0.151 |
| BMS-708163 | 10.180 | 9.825 | 10.491 | 9.302 | 9.391 | 10.425 |
| BMS-754807 | 9.531 | 9.417 | 9.762 | 9.676 | 9.806 | 10.462 |
| Bortezomib | -7.640 | -7.583 | -7.734 | -7.467 | -7.499 | -7.763 |
| Bosutinib | 6.880 | 7.131 | 7.213 | 1.716 | 1.709 | 1.934 |
| BX-795 | 14.261 | 14.985 | 14.692 | 10.538 | 10.499 | 9.238 |
| Camptothecin | -3.435 | -2.336 | -3.815 | -5.449 | -5.630 | -7.044 |
| CCT018159 | 3.571 | 3.628 | 3.704 | 3.380 | 3.372 | 3.447 |
| CEP-701 | 5.531 | 6.983 | 5.760 | 1.229 | 1.013 | -0.386 |
| CGP-60474 | -4.974 | -4.931 | -5.082 | -5.107 | -5.124 | -5.258 |
| CI-1040 | 1.452 | 1.891 | 1.451 | 1.137 | 1.026 | 0.402 |
| Cisplatin | 1.372 | 1.217 | 1.559 | 2.240 | 2.287 | 2.259 |
| CMK | 4.290 | 4.010 | 3.707 | 3.711 | 3.664 | 3.538 |
| Cyclopamine | 4.286 | 4.585 | 4.394 | 4.288 | 4.256 | 4.008 |
| Cytarabine | -0.520 | 0.303 | -0.362 | -0.818 | -0.911 | -1.998 |
| Dasatinib | 0.125 | -0.260 | -1.146 | -1.700 | -1.808 | -3.447 |
| DMOG | 10.645 | 10.618 | 10.696 | 8.047 | 8.100 | 8.172 |
| Docetaxel | -8.386 | -8.319 | -7.975 | -9.070 | -9.040 | -8.972 |
| Doxorubicin | -6.808 | -6.943 | -6.807 | -6.527 | -6.522 | -6.463 |
| EHT-1864 | 8.782 | 9.264 | 8.381 | 7.899 | 7.758 | 6.505 |
| Embelin | 0.310 | 0.302 | 0.411 | 0.240 | 0.248 | 0.276 |
| Epothilone-B | -9.296 | -9.467 | -8.538 | -8.951 | -8.897 | -8.008 |
| Erlotinib | 6.013 | 5.646 | 5.972 | 5.466 | 5.584 | 6.328 |
| Etoposide | -1.816 | -1.994 | -1.423 | -2.773 | -2.729 | -2.581 |
| FH535 | 0.273 | 0.260 | 0.327 | 0.330 | 0.334 | 0.395 |
| GDC-0449 | 7.859 | 7.916 | 8.289 | 6.623 | 6.605 | 7.237 |
| GDC0941 | 4.144 | 3.908 | 4.198 | 3.556 | 3.686 | 4.291 |
| Gefitinib | 2.135 | 2.098 | 1.793 | 2.009 | 1.917 | 0.902 |
| Gemcitabine | -9.328 | -8.190 | -9.118 | -4.165 | -4.582 | -7.590 |
| GSK-650394 | 1.520 | 1.410 | 2.533 | 0.690 | 0.658 | 1.291 |
| GW843682X | -5.203 | -5.190 | -5.209 | -5.121 | -5.123 | -5.193 |
| Imatinib | 5.356 | 5.325 | 5.606 | 5.303 | 5.379 | 5.801 |
| JNJ-26854165 | 4.058 | 4.015 | 4.049 | 4.221 | 4.249 | 4.350 |
| JNK-9L | -0.889 | -0.657 | -0.381 | -2.132 | -2.135 | -2.226 |
| JNK-Inhibitor-VIII | 5.455 | 5.492 | 5.304 | 6.008 | 5.964 | 5.620 |
| KIN001-135 | 3.743 | 3.669 | 3.700 | 5.008 | 5.028 | 4.979 |
| Lenalidomide | 5.430 | 5.466 | 5.325 | 4.632 | 4.573 | 4.316 |
| LFM-A13 | 3.774 | 2.925 | 4.269 | 4.570 | 4.697 | 5.828 |
| Metformin | 10.317 | 10.410 | 10.273 | 10.158 | 10.135 | 9.827 |
| Methotrexate | 4.005 | 3.956 | 4.184 | 3.638 | 3.709 | 4.137 |
| Midostaurin | 10.990 | 11.116 | 12.319 | 3.818 | 3.964 | 4.731 |
| Mitomycin-C | -3.170 | -3.217 | -3.040 | -3.132 | -3.146 | -3.174 |
| MS-275 | 5.311 | 5.590 | 5.593 | 4.508 | 4.654 | 5.233 |
| Nilotinib | 3.389 | 3.425 | 3.509 | 3.273 | 3.261 | 3.336 |
| NSC-87877 | 8.442 | 8.324 | 8.571 | 7.546 | 7.592 | 8.058 |
| Nutlin-3a | 6.905 | 7.271 | 6.707 | 6.521 | 6.416 | 5.499 |
| NVP-TAE684 | 4.575 | 4.264 | 6.772 | 8.349 | 8.818 | 15.364 |
| OSI-906 | 7.233 | 7.071 | 7.270 | 7.926 | 7.997 | 8.276 |
| Paclitaxel | -6.795 | -6.744 | -6.691 | -7.149 | -7.148 | -7.255 |
| Parthenolide | 4.505 | 5.235 | 4.232 | 4.348 | 4.063 | 1.353 |
| Pazopanib | 7.108 | 6.752 | 7.179 | 6.474 | 6.495 | 7.159 |
| PD-0325901 | -3.240 | -2.363 | -2.924 | -1.088 | -1.227 | -2.052 |
| PD-0332991 | 8.893 | 9.997 | 8.886 | 4.053 | 3.731 | 1.972 |
| PD-173074 | 6.803 | 6.827 | 6.795 | 5.693 | 5.654 | 5.216 |
| PF-02341066 | 3.863 | 3.865 | 3.833 | 4.096 | 4.092 | 4.060 |
| PF-4708671 | 2.118 | 1.864 | 2.015 | 2.857 | 2.856 | 3.168 |
| PF-562271 | 3.696 | 3.759 | 3.729 | 2.744 | 2.712 | 2.614 |
| PHA-665752 | 4.523 | 4.525 | 4.520 | 4.475 | 4.472 | 4.457 |
| Pyrimethamine | 2.817 | 2.156 | 3.798 | 2.183 | 2.457 | 4.660 |
| Rapamycin | -1.353 | -2.340 | -1.261 | -5.529 | -5.146 | -5.014 |
| RO-3306 | 7.685 | 7.980 | 7.594 | 6.588 | 6.420 | 5.512 |
| Roscovitine | 10.322 | 11.479 | 10.430 | 6.069 | 5.843 | 4.194 |
| Salubrinal | 4.955 | 5.105 | 4.858 | 4.031 | 4.025 | 3.655 |
| SB-216763 | 7.083 | 7.119 | 7.166 | 6.697 | 6.693 | 6.855 |
| Shikonin | -3.761 | -3.737 | -3.648 | -3.867 | -3.854 | -3.742 |
| SL-0101-1 | 4.495 | 4.339 | 3.952 | 4.690 | 4.632 | 3.376 |
| Sorafenib | 8.487 | 8.561 | 8.427 | 6.946 | 6.904 | 6.964 |
| S-Trityl-L-cysteine | -0.673 | -0.549 | -0.392 | -0.975 | -0.931 | -1.063 |
| Sunitinib | 4.054 | 4.085 | 4.075 | 3.697 | 3.690 | 3.690 |
| Temsirolimus | 5.741 | 5.632 | 5.950 | 2.622 | 2.585 | 1.567 |
| Thapsigargin | -3.455 | -3.957 | -3.287 | -7.534 | -7.557 | -8.403 |
| Tipifarnib | -2.847 | -2.838 | -2.351 | -3.444 | -3.465 | -3.334 |
| TW-37 | -0.261 | -0.260 | -0.258 | -0.252 | -0.252 | -0.254 |
| Vinblastine | -5.151 | -4.798 | -4.963 | -6.832 | -6.830 | -6.948 |
| Vinorelbine | -5.984 | -6.021 | -5.758 | -7.483 | -7.473 | -7.476 |
| Vorinostat | 6.725 | 7.060 | 6.974 | 5.140 | 5.233 | 5.150 |
| VX-680 | 1.567 | 1.922 | 1.988 | 0.121 | 0.087 | -0.039 |
| VX-702 | 6.691 | 6.737 | 6.772 | 6.527 | 6.535 | 6.585 |
| WH-4-023 | 0.922 | 0.679 | 0.300 | 1.829 | 1.878 | 1.479 |
| WO2009093972 | 0.610 | 1.252 | 0.914 | 1.170 | 0.888 | -1.824 |
| WZ-1-84 | 1.476 | 0.438 | 1.176 | 2.987 | 3.016 | 3.837 |
| X17-AAG | -0.958 | -0.773 | -0.644 | -3.357 | -3.410 | -3.471 |
| X681640 | 4.285 | 4.512 | 4.430 | 3.058 | 3.007 | 2.619 |
| XMD8-85 | 2.982 | 3.388 | 3.453 | 2.014 | 1.982 | 1.740 |
| ZM-447439 | 4.349 | 5.218 | 4.352 | 4.350 | 4.090 | 2.335 |

**Table S7**. Primers used in the study.

| **Primer** | **Sequence (5' - 3')** |
| --- | --- |
| **Clone primers** |  |
| CDS_PIGR_F^†^ | CGGAATTCGCCACCATGCTGCTCTTCGTGCTCACCT |
| CDS_PIGR_R^†^ | CCAAGCTTCTAGGCTTCCTGGGGGCC |
| CDS_LAMB3_F | AGCTCGAGGCCACCATGAGACCATTCTTCCTCTTGTGTT |
| CDS_LAMB3_R | TAGAATTCTCACTTGCAGGTGGCATAGTAGAG |
| GFP_PIGR_F | CGGAATTCGCCACCATGCTGCTCTTCGTGCTCACCT |
| GFP_PIGR_R | CCCCCCGGGCGGCTTCCTGGGGGCCGTC |
| FLAG_REST_F | GCAAGCTTGCCACCATGGCCACCCAGGTAATGG |
| FLAG_REST_R | GGTCTCGAGCTCCTGCCCTTGAGCTGC |
| PIGR_S673A_F | AACGTCGACCGAGTTGCAATCAGAAGCTACA |
| PIGR_S673A_R | TGTAGCTTCTGATTGCAACTCGGTCGACGTT |
| PIGR_T679A_F | ATCAGAAGCTACAGGGCAGACATTAGCATGT |
| PIGR_T679A_R | ACATGCTAATGTCTGCCCTGTAGCTTCTGAT |
| PIGR_S735A_F | AAGGCAAAAAGGTCAGCCAAGGAGGAAGCCG |
| PIGR_S735A_R | CGGCTTCCTCCTTGGCTGACCTTTTTGCCTT |
| PIGR_S743A_F | AAGCCGAGATGGCCTTCAAAGACTTCCTGCT |
| PIGR_S743A_R | AGCAGGAAGTCTTTGAAGGCCATCTCGGCTT |
| Linearize_pGL3_F | TGCGATCTAAGTAAGCTTGG |
| Linearize_pGL3_R | GATCTCGAGCCCGGGCTAGC |
| Promoter_LAMB3_F | GCTAGCCCGGGCTCGAGATCACTTGGAAATGATCAGTGTC |
| Promoter_LAMB3_R | CCAAGCTTACTTAGATCGCAGGGGGTCTCCCCTCCCGGCT |
| **RTq-PCR primers** |  |
| PIGR_F | GGCTACGTCTCCAGCAAATATG |
| PIGR_R | CCTCGGCTATTGATGCCCAG |
| LAMB3_F | GCAGCCTCACAACTACTACAG |
| LAMB3_R | CCAGGTCTTACCGAAGTCTGA |
| GAPDH_F | CTGGGCTACACTGAGCACC |
| GAPDH_R | AAGTGGTCGTTGAGGGCAATG |

^†^suffix “F” represents forward primer; “R” represents reverse primer.
